# Mechano-Induced Synthesis of Polyethylene Glycols α,ω-DiSubstituted with 4-(PAH)-1*H*-1,2,3-Triazoles in Presence of *In Situ*-Generated Bronze Microparticles as Catalyst

**DOI:** 10.3390/molecules31020270

**Published:** 2026-01-13

**Authors:** Mohammed S. Mohammed, Igor S. Kovalev, Vadim A. Platonov, Sougata Santra, Zhuo Wang, Grigory V. Zyryanov, Valery N. Charushin

**Affiliations:** 1 Department of Organic and Biomolecular Chemistry, Chemical Engineering Institute, Ural Federal University, 19, Mira Str., Yekaterinburg 620002, Russia; samirmohammed422@gmail.com (M.S.M.); vplatonov10@mail.ru (V.A.P.); g.v.zyrianov@urfu.ru (G.V.Z.); charushin@ios.uran.ru (V.N.C.); 2 I. Ya. Postovsky Institute of Organic Synthesis of RAS (Ural Division), 22/20, S. Kovalevskoy/Akademicheskaya Str., Yekaterinburg 620137, Russia; 3 State Key Laboratory of Chemical Resource Engineering, College of Chemistry, Beijing Advanced Innovation Center for Soft Matter Science and Engineering, Beijing University of Chemical Technology, Beijing 100029, China; wangzhuo77@mail.buct.edu.cn

**Keywords:** mechanochemistry, triazoles, polyethylene glycols, bronze microparticles, copper

## Abstract

A method for the mechanochemical CuAAC click reaction (Cu^+^-promoted azido–alkyne cycloaddition) in the presence of bronze powder/copper beads without the use of a pre-introduced catalyst and ligands with greener prospect is presented. A new type of tri-, tetra-, and penta-ethylene glycols (PEGs) α,ω-disubstituted with 4-(PAH)-1*H*-1,2,3-triazole moieties has been synthesized by means of solvent-free click reaction in the planetary ball-milling in absence of a pre-introduced Cu(I) catalyst. The reaction afforded the above-mentioned compounds at room temperature in as short as 3 h in up to 96% yields and with *E*-factor values as low as 0.38. For the comparison, some of the key compounds were obtained by the conventional click synthesis in DMF solution. The compounds obtained were synthesized for the first time and can be considered as representative examples of bola-type chemosensors for the detection of electron-deficient species. This work presents a method for catalyzing a click reaction using bronze microparticles that are formed in situ during powder milling. This heterophase catalyst has been shown to be efficient and inexpensive and is suitable for green chemistry methods under solvent-free ball-milling conditions.

## 1. Introduction

In the past decade, there has been a persistent growth of interest in mechanochemical reactions through milling or grinding [1], whereas the application of mechanochemistry to inorganic materials (e.g., minerals and ores) has been known since antiquity [2]. This tremendous rise in interest in milling or grinding synthesis [3] has enormous expansion of mechanochemical procedures towards synthesis of organic materials [4,5,6], supramolecular [7] and coordination ones [8]. The context of *Green Chemistry*, the solvent-free technique, is the most often considered improvement in reaction design which can be offered by mechanochemistry because the last one excludes the bulk solvent use [9,10,11]. Ball-milling chemistry is a practical method, which is considered as environmentally friendly, and this technique has found interest in synthetic organic chemistry [5,12,13,14]. Among the reported examples, we can mention nitrone synthesis [15], reductive benzylation of malonitrile [16], protection of amines [17], Michael additions [18], Glaser coupling [19], nucleoside chemistry [20], and peptide coupling [21]. Regarding click reaction, the copper(I)-catalyzed azide–alkyne cycloaddition (CuAAC) represents a prime example of click chemistry. Click chemistry describes “a set of near-perfect” reactions [22,23,24]. Click reactions affording 1,2,3-triazoles rapidly became important for simple and robust binding of versatile molecules and for the building of stable structures [25]. At the same time, the 1,2,3-triazoles became the heterocycle of choice in drug discovery, due to their favorable pharmacokinetic and safety profiles, hydrogen-bonding capability, moderate dipole moment, and rigidity and stability under in vivo conditions [26,27]. Several other click reactions have been introduced with ball-milling technique [28,29,30,31,32].

Poly(ethylene glycol) (PEG) is gaining widespread attention for use in preparation of click reaction. PEG has tremendous applications in biological contexts and for industrial uses [33,34,35]. For instance, PEG-appended 1,2,3-triazole was prepared via click reaction between PEG-azide and phenylacetylene and used as an efficient precatalyst for palladium-catalyzed Suzuki–Miyaura and copper-free Sonogashira reaction in water [36]. Wang and co-authors developed parallel synthesis of PEG-6000-supported 1,2,3-triazole derivatives by means of Cu-catalyzed click reaction between PEG-6000-appended azide and various mono- and disubstituted alkynes, including acetylene with the following cleavage of PEG support to afford substituted 1,2,3-triazoles [37]. Very recently, PEGylated water-soluble 1,2,4-triazoles were prepared from methyl 5-(chloromethyl)-1-aryl-1*H*-1,2,4-triazole-3-carboxylates and polyethyleneglycols as anti-inflammatory agents and some of them exhibited better in vitro and in vivo bioactivity than Indomethacin [38].

Most of the reported polycyclic aromatic hydrocarbon (PAH) podands could be considered as ionophores or probes, including those based on polycyclic aromatic hydrocarbons (PAHs) such as pyrene [39,40,41,42]. Due to its known photoreactivity [39,43,44,45,46], pyrene has more promising photophysical properties than other compounds, such as intensive monomeric and excimeric emission [47,48,49,50]. Very few bola-type pyrene-based podands have been reported so far [48,51]. The introduction of PEG moieties in these kinds of architectures provides both the better water solubility and, importantly, the ability of host molecules to adopt their conformation according to guest geometry. Therefore, these bola-type structures would have the ability to spectral changes in case of combining with pyrene moieties [52,53,54]. Because of these unique qualities and features, pyrene-poly(ethylene glycol) chemosensors have already attracted wide attention. For instance, Suzuki et al. reported an intramolecular excimer-to-monomer fluorescence of pyrene-appended non-cyclic crown ethers and their fluorescence responses towards alkali and alkaline earth metal cations [55]. Additionally, optically active pyrene-containing podands exhibiting polarized luminescence were reported [56].

**Scheme 1:**
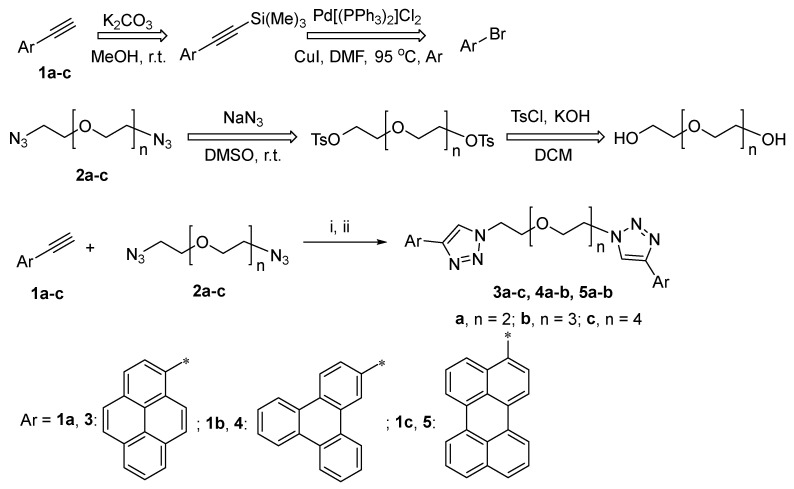
Synthesis of PEG-linked bis-4-(PAH)-1,2,3-triazoles **3**–**5** with retrosynthetic schemes for precursors **1**–**2** *Reagents and conditions:* [*i*, ball milling, 500 rpm, 3 h]; *ii*, sodium ascorbate, CuSO_4_ × 5H_2_O (0.40 equiv.), DMF, 65 °C, Ar. Asterisk symbol (*)-Aryl residue attachement’s position.

Owing to the importance of the above-mentioned compounds in various fields, in this work, we wish to report a mechano-induced solvent- and catalyst-free approach to the new bola-type architectures, namely polyethylene glycols α,ω-disubstituted with 4-(PAH)-1*H*-1,2,3-triazoles (Scheme 1). This work presents a method for catalyzing a click reaction using bronze microparticles that are formed in situ during ball-milling of the bronze powder. Thus formed heterophase catalyst has been shown to be efficient and inexpensive and is suitable for green chemistry methods under solvent-free ball-milling conditions.

## 2. Results and Discussion

In order to synthesize a series of PEG-linked bis-4-(PAH)-1,2,3-triazoles, such as **3a**–**c**, **4a**,**b** and **5a**,**b**, in a frame of current research, two approaches were investigated (Scheme 1)—namely, “click” reactions between azide-substituted PEG with a PAH-containing ethynyl component in solution or under ball-milling conditions. 1-Ethynylpyrene **1a** was prepared as an ethynyl component from 1-bromopyrene, according to the previously reported method [57]; several PEG-based azido components **2a**–**c** have been prepared as azido components, according to the previously reported method [58], starting from the corresponding PEG bis (tosylates).

**Figure 1:**
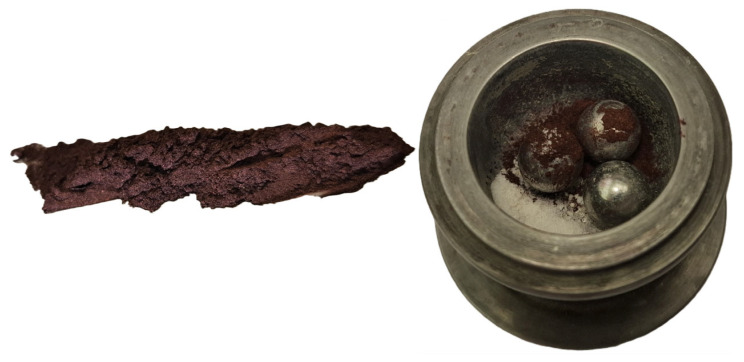
Photos of bronze powder (**left**) and the ball-milling setup with loading (**right**).

To achieve low *E*-factors of the reaction, we assumed the reaction to be carried out first between azido- and ethynyl components without using any solvent, namely, under mechanochemical conditions. The compliance of this mechanochemical protocol with the criteria of green reaction is established by the green metrics, *E*-factors. In 1992, the E-factor, or environmental impact factor, was introduced by Roger Sheldon [59,60]. These metrics help to quantify the amount of waste generated per kilogram of product. Conventionally, the *E*-factor is a quantitative tool used to evaluate the quality of an organic process based on the generated waste. Previously, several successful examples of mechanochemically induced click reactions were reported for the synthesis of 1,2,3-triazoles [28,29,30,31,32]. Moreover, based on our previous findings [32], reaction appeared to be successful in the absence of pre-introduced copper(I) salts as catalysts. According to the data of the reaction optimization studies (Table 1), the optimal conditions were achieved by reacting 1-ethynylpyrene **1a** with the corresponding azido component **2a** by milling in a stainless-steel 50 mL milling jar with four 10 mm milling copper balls in the presence of abrasive powder, such as silica gel (Kieselgel 60 0.04–0.063 mm Macherey-Nagel) or bronze (20% tin) powder at 500 rpm. As a result, 1-ethynylpyrene **1a** and the corresponding azido components **2a**–**c** were reacted readily to afford the target 1,2,3-triazole-appended PEGs **3a**–**c** (Table 1, entries 1–2, 4–5, 7–8). It is worth mentioning that the reaction was also carried out in the absence of copper milling balls, namely, under ball milling in a stainless-steel 50 mL milling jar with four 10 mm milling stainless-steel balls at 500 rpm for 3 h in the presence of bronze powder, and the target PEGs **3a**–**c** were isolated in similar yields (Table 1, entries 3, 6, 9); see Figure 1.

To confirm the effectiveness of “click” reactions under mechanochemical conditions, the second approach was studied. To do this, the reaction between **1a** and **2a**–**c** was carried out according to previously developed conditions for the CuAAC reactions, namely, upon the reaction between azido and ethynyl components by means heating in a solution of DMF for 16 h in the presence of cuprous sulfate, obtained *in situ* from NaOH and ascorbic acid, and copper(II) sulfate pentahydrate. The results are presented in Table 1, entries 10–12 (Procedure D (see Section 3.3. in the experimental part for more details)).

Based on the data obtained (Table 1), the mechano-synthesis using copper or steel balls in the presence of bronze powder seems to be the most effective for obtaining compounds **3**, whereas in the reaction in solution in all the cases, the yields were comparatively lower (Table 1, entries 10–12). Another undoubted advantage of the mechano-synthesis-based approach is a significant reduction in reaction time (3 h vs. 16 h), room temperature reaction, the absence of a solvent, and, most importantly, the absence of pre-loaded copper(I) catalyst as well as ligands. The highest yield observed in case of using bronze powder and stainless-steel balls (Procedure C (see Section 3.3. in the experimental part for more details)) can be explained by the transition of soft bronze particles into a finely dispersed catalytically active state when grinding with hard steel balls. This procedure has proven to be the best, also having the advantage of using standard mill equipment made of common materials (steel) and only bronze powder as an additive.

This effect was not achieved when using relatively soft copper balls, even in the presence of an abrasive additive (silica gel, see Procedure A) or in the presence of an additive in the form of bronze powder (Procedure B). In addition to the reduced yields, procedures A–B have the disadvantage of requiring the use of copper balls. Finally, *E*-factors for each reaction were calculated, and for the ball-milling-based procedures lowest *E*-factors values were obtained.

In the ^1^H NMR spectrum of compound **3b** (See Supporting Information, Figure S2), signals of the PEG bridge methylenes’ protons are observed in the range of 3.48–4.46 ppm as triplets. Signals of the protons of the polyaromatic fragment (1-pyrenyl) are observed as a multiplet in the region of 7.95–8.20 ppm. Characteristic for this compound is the signal of the 2-H proton of 1-pyrenyl with a chemical shift near 8.65 ppm in the form of dublet. The signal of the C-H proton of the triazole is not isolated (mixed with the peaks of the signals of the pyrenyl residue and is indistinguishable) and can only be confirmed by integration. ^1^H and 13C NMR spectra of the compounds **3**-**5** can be found in Supporting Information.

As a next step, to expand the range of PAH substituents in bola-type compounds, triphenylene- and perylene-substituted 1,2,3-triazoles containing PEGs were prepared in the same way, namely, via a “click” reaction between azido components **2a**–**b** and corresponding ethynyl components **1b**–**c** (Scheme 1, Table 2). The PAH-based ethynyl components were synthesized according to a described procedure [61]. As a result, the target PAH-based bola-type compounds **4**, **5** were obtained in up to 96% yields. Similarly to the above-mentioned results, the best conditions were found to be the ones using stainless-steel milling balls and bronze powder as an additive (Table 2, entries 6 and 12, Procedure C).

### Proposed Mechanism of Click-Reaction Catalysis in the Presence of Bronze Powder or Copper Balls

**Scheme 2:**
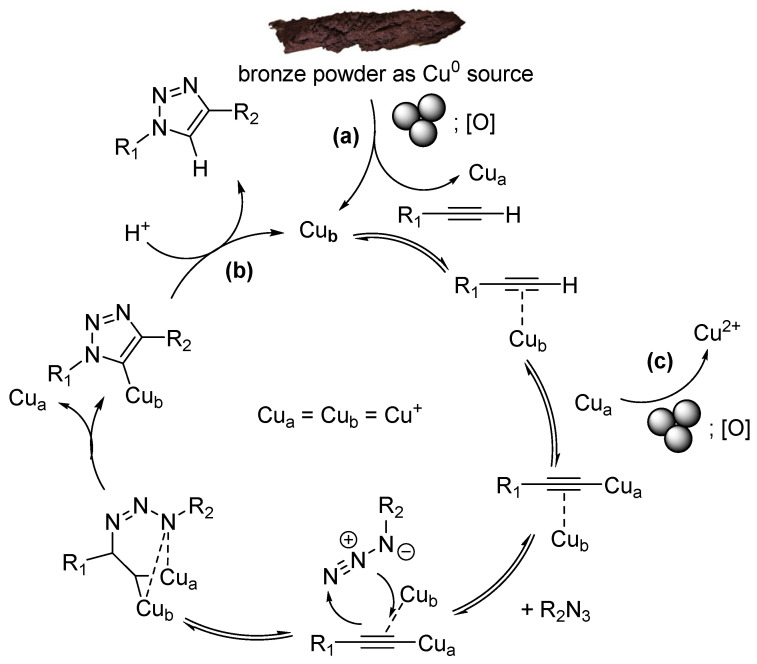
CuAAC mechanism.

This highly effective procedure can be explained as follows (Scheme 2) [62]. Under ball-milling/grinding conditions, the surfaces of the milling copper balls and/or bronze particles are continuously cleaned mechanically. On the surface layers of both the balls and the particles, copper atoms are continuously oxidized by atmospheric oxygen to the Cu^(+1)^ state (Scheme 2, (a)). Once a catalytic center forms on the surface, it catalyzes the CuAAC reaction cycle (Scheme 2, (b)) until it oxidizes further to a Cu^(+2)^ state (Scheme 2, (c)). Subsequently, the ball milling/grinding mechanically renews the surface of the balls and particles, removing the Cu^(+2)^ atoms, and the cycle Cu^(0)^ → Cu^(+1)^ → Cu^(+2)^ → Cu^(0)^ repeats. Interestingly, Cu^(+2)^ may be involved in the formation of copper acetylides (as Cu_a_ substitute in Scheme 2) accelerating formation of triazoles rather than slowing it [63].

Furthermore, it can be assumed that copper/bronze microparticles are released from the copper or bronze surfaces into the reaction mixture due to the grinding. The same cycle of copper oxidation states, Cu^(0)^ → Cu^(+1)^ → Cu^(+2)^ → Cu^(0)^, is then realized directly within the reaction medium. Both the number of such particles and their surface area increase over time. Continuously, the grinding/ball milling exposes deeper layers of copper atoms, which then participate in the catalytic process. These synergistic effects cause the click reaction to proceed with significant acceleration and within a short time.

## 3. Materials and Methods

### 3.1. General Information

All reagents were purchased from commercial sources and used without further purification. Silica gel 60 (Kieselgel 60, 230–400 mesh, MACHEREY-NAGEL GmbH & Co. KG, Dueren, Germany) was used for the column chromatography. NMR spectra were recorded on a Bruker Avance-400 spectrometer (Bruker Corporation, Rheinstetten, Germany), 298 K, digital resolution ± 0.01 ppm, using TMS as internal standard. Ball-milling experiments were carried out on a Retsch PM 100 CM ball mill (Retsch GmbH, Haan, Germany) in a 50 mL stainless-steel milling jar. Elemental analyses were performed on a PE 2400 II CHN-analyzer (Perkin Elmer Inc., Waltham, MA, USA). Mass spectrometry data were acquired using an Agilent 6545 Q-TOF LC-MS (Agilent Technologies, Santa Clara, CA, USA) with electrospray ionization.

### 3.2. Safety Statement

Since we used organic azido derivatives in the ball-milling process, it is worth mentioning the danger of this process exists, due to the potential for explosion, so caution is advised. Compounds **2** were previously tested by us for sensitivity to impact and friction and proved to be inert towards mechanical actions.

### 3.3. Preparation of α,ω-Disubstituted Polyethylene Glycols

Procedure A. Corresponding PAH-ethynyl (2.0 mmol) and azido components (1.05 mmol), 10 mg of additive (Silica Gel Kiselgel 60 0.04–0.063 mm Macherey-Nagel) were placed in a steel jar with five copper balls and agitated at 500 rpm for 3 h. Reaction progress was monitored by TLC. Upon completion of the reaction, the reaction product was extracted with CH_2_Cl_2_ (3 × 25 mL) and the extract was evaporated in vacuo. The residue was flash-chromatographed (silica gel, impurities were eluted with toluene, the product was eluted with ethyl acetate). The fractions containing the product were collected and evaporated in vacuo.

Procedure B. According to Procedure A with bronze powder (200 mg) as additive.

Procedure C. According to Procedure A with four steel balls only and bronze powder (200 mg) as additive.

Procedure D. In a 50 mL flask, sodium hydroxide (0.80 mmol), ascorbic acid (1 mmol), and copper(II) sulfate pentahydrate (0.40 mmol) were sequentially dissolved in water (3 mL). The resulting yellow solution was evaporated to dryness in vacuo and the residue was suspended in 3 mL of DMF. A solution of the corresponding ethynyl derivative (2.40 mmol) and azido derivative (1.05 mmol) in DMF were added to the resulting milky suspension. The flask was purged with argon and stirred under argon at 65 °C for 16 h. Upon completion of the reaction, the reaction product was extracted with CH_2_Cl_2_ (3 × 25 mL) and the extract was evaporated in vacuo. The residue was flash-chromatographed (silica gel, impurities were eluted with toluene, the product was eluted with ethyl acetate). The fractions containing the product were collected and evaporated in vacuo.

1,2-Bis(2-(4-(pyren-1-yl)-1*H*-1,2,3-triazol-1-yl)ethoxy)ethane (**3a**)

Procedure A. Yield 385 mg, 59%; B. Yield 595 mg, 91%; C. Yield 550 mg, 84%; D. Yield 262 mg, 40%. ^1^H NMR (400 MHz, CDCl_3_, δ, ppm): 3.66 (t, 4H, 2 × CH_2_O), 3.97 (t, 4H, 2 × CH_2_O), 4.58 (t, 4H, 2 × CH_2_O), 7.93–8.03 (m, 10H, Pyr), 8.09–8.14 (m, 8H, Pyr + C_2_N_3_H), 8.58 (d, 2H, Pyr). ^13^C NMR (100 MHz, DMSO-*d*_6_, *δ*, ppm): 50.3, 60.4, 69.4, 70.4, 124.6, 124.6, 124.8, 125.0, 125.4, 126.0, 127.0, 127.2, 127.7, 128.0, 128. 3, 130.7, 131.2, 131.3. Anal. Calcd. For C_42_H_32_N_6_O_5_: C 77.28%, H 4.94%, N 12.87%; Found: C 77.17%, H 5.07%, N 12.80%. ESI-MS, *m*/*z*, (%): Calcd. For [C_42_H_32_N_6_O_5_ + H]^+^ = 701.2507; Found: 701.2514, (100).

1,1′-(((Oxybis(ethane-2,1-diyl))bis(oxy))bis(ethane-2,1-diyl))bis(4-(pyren-1-yl)-1*H*-1,2,3-triazole) (**3b**)

Procedure A. Yield 555 mg, 78%; B. Yield 460 mg, 65%; C. Yield 640 mg, 90%; D. Yield 500 mg, 70%. ^1^H NMR (400 MHz, CDCl_3_, δ, ppm): 3.55 (m, 4H, 2 × CH_2_O), 3.56 (m, 4H, 2 × CH_2_O), 3.69 (t, 4H, 2 × CH_2_O), 4.45 (t, 4H, 2 × CH_2_O), 7.95–8.07 (m, 10H, Pyr), 8.13–8.19 (m, 8H, Pyr + C_2_N_3_H), 8.64 (d, 2H, Pyr). ^13^C NMR (100 MHz, DMSO-*d*_6_, *δ*, ppm): 50.1, 69.2, 70.4, 124.1, 124.7, 124.8, 125.0, 125.1, 125.3, 125.4, 125.4, 126.1, 127.1, 127.3, 127.8, 128.1, 128.2, 128.5, 129.0, 130.8, 131.2, 131.3, 137.9, 147.1. Anal. Calcd. For C_45_H_38_N_6_O_3_: C 76.04%, H 5.39%, N 11.82%; Found: C 75.92%, H 5.48%, N 11.75%. ESI-MS, *m*/*z*, (%): Calcd. For [C_45_H_38_N_6_O_3_ + H]^+^ = 711.3078; Found: 711.3072, (100).

1,14-Bis(4-(pyren-1-yl)-1*H*-1,2,3-triazol-1-yl)-3,6,9,12-tetraoxateradecane (**3c**)

Procedure A. Yield 500 mg, 68%; B. Yield 555 mg, 75%; C. Yield 645 mg, 87%; D. Yield 520 mg, 70%. ^1^H NMR (400 MHz, CDCl_3_, *δ*, ppm): 3.32–3.36 (m, 4H, 2 × CH_2_O), 3.37–3.38 (m, 8H, 4 × CH_2_O), 3.74 (t, 4H, 2 × CH_2_O), 4.53 (t, 4H, 2 × CH_2_O), 7.95–8.00 (m, 2H, Pyr), 8.02–8.07 (m, 8H, Pyr + C_2_N_3_H), 8.13–8.16 (m, 6H, Pyr), 8.19–8.21 (m, 2H, Pyr), 8.67 (d, 2H, Pyr). ^13^C NMR (100 MHz, DMSO-*d*_6_, *δ*, ppm): 49.1, 52.4, 69.1, 69.2, 123.1, 123.6, 123.7, 124.0, 124.2, 124.4, 125.0, 126.0, 126.2, 126.6, 127.0, 127.3, 129.7, 130.0, 130.2, 146.0. Anal. Calcd. For C_46_H_40_N_6_O_4_: C 74.58%, H 5.44%, N 11.34%; Found: C 74.77%, H 5.21%, N 11.22%. ESI-MS, *m*/*z*, (%): Calcd. For [C_46_H_40_N_6_O_4_ + H]^+^ = 741.3184; Found: 741.3178, (100).

1,2-Bis(2-(4-(triphenylen-2-yl)-1*H*-1,2,3-triazol-1-yl)ethoxy)ethane (**4a**)

Procedure A. Yield 405 mg, 69%; B. Yield 514 mg, 88%; C. Yield 472 mg, 80%; D. Yield 350 mg, 50%. ^1^H NMR (400 mHz, DMSO-*d*_6_, *δ*, ppm): 3.61 (s, 4H, 2 × CH_2_O), 3.92 (t, 4H, 2 × CH_2_O), 4.61 (t, 4H, 2 × CH_2_O), 7.69 (m, 8H, C_18_H_11_), 8.16 (m, 2H, C_18_H_11_), 8.73–8.89 (m, 12H, C_18_H_11_), 9.18 (s, 2H, C_2_N_3_H). ^13^C NMR (100 MHz, DMSO-*d*_6_, *δ*, ppm): 49.8, 68.7, 69.6, 119.5, 122.4, 123.52, 123.55, 123.59, 123.62, 124.32, 124.45, 127.57, 127.58, 127.63, 127.77, 128.59, 128.96, 129.08, 129.37, 129.87, 146.17. Anal. Calcd. For C_46_H_36_N_6_O_2_: C 78.39%, H 5.15%, N 11.92%; Found: C 78.30%, H 5.25%, N 11.84%. ESI-MS, *m*/*z*, (%): Calcd. For [C_46_H_36_N_6_O_2_ + H]^+^ = 705.2973; Found: 705.2979, (100).

1,1′-(((Oxibis(ethane-2,1diyl))bis(oxy))bis(ethane-2,1-diyl))bis(4-(triphenylen-2-yl)-1*H*-1,2,3-triazole) (**4b**)

Procedure A. Yield 530 mg, 71%; B. Yield 560 mg, 75%; C. Yield 720 mg, 96%; D. Yield 635 mg, 85%. ^1^H NMR (400 mHz, DMSO-*d*_6_, *δ*, ppm): 3.50–3.55 (m, 8H, 4 × CH_2_O), 3.87 (t, 4H, 2 × CH_2_O), 4.59 (t, 4H, 2 × CH_2_O), 7.69–7.73 (m, 8H, C_18_H_11_), 8.17–8.19 (m, 2H, C_18_H_11_), 8.76–8.90 (m, 12H, C_18_H_11_), 9.18 (s, 2H, C_2_N_3_H). ^13^C NMR (100 MHz, DMSO-*d*_6_, *δ*, ppm): 67.65, 68.88, 69.97, 95.40, 115.42, 121.82, 122.45, 123.88, 124.28, 124.90, 125.15, 125.50, 126.39, 127.10, 127.25, 127.60, 127.73, 128.01, 130.13, 130.34, 130.70, 130.89, 147.02, 158.61. Anal. Calcd. For C_48_H_40_N_6_O_3_: C 76.98%, H 5.38%, N 11.22%; Found: C 76.88%, H 5.29%, N 11.20%. ESI-MS, *m*/*z*, (%): Calcd. For [C_48_H_40_N_6_O_3_ + H]^+^ = 749.3235; Found: 749.3240, (100).

1,2-Bis(2-(4-(perylen-3-yl)-1*H*-1,2,3-triazol-1-yl)ethoxy)ethane (**5a**)

Procedure A. Yield 360 mg, 48%; B. Yield 525 mg, 70%; C. Yield 585 mg, 78%; D. Yield 185 mg, 25%. ^1^H NMR (400 mHz, DMF-*d_7_*, *δ*, ppm): 4.09 (s, 4H, 2 × CH_2_O), 4.45 (t, 4H, 2 × CH_2_O), 5.14–5.15 (t, 4H, 2 × CH_2_O), 7.89–7.93 (m, 6H, C_20_H_13_), 8.24 (m, 6H, C_20_H_13_), 8.90–9.04 (m, 10H, C_20_H_13_), 9.34 (s, 2H, C_2_N_3_H). ^13^C NMR (100 MHz, DMSO-*d*_6_, *δ*, ppm): 50.28, 69.90, 70.66, 121.04, 121.36, 121.53, 121.55, 125.05, 126.51, 127.35, 127.41, 127.98, 128.37, 128.44, 128.87, 129.02, 129.59, 130.13, 131.45, 131.52, 131.82, 132.57, 135.27, 146.45, 162.54. Anal. Calcd. For C_50_H_36_N_6_O_2_: C 79.77%, H 4.82%, N 11.16%; Found: C 79.67%, H 4.91%, N 11.08%. ESI-MS, *m*/*z*, (%): Calcd. For [C_48_H_40_N_6_O_3_ + H]^+^ = 753.2973; Found: 753.2964, (100).

1,1′-(((Oxibis(ethane-2,1-diyl))bis(oxy))bis(ethane-2,1-diyl))bis(4-(perylen-3-yl)-1*H*-1,2,3-triazole) (**5b**)

Procedure A. Yield 550 mg, 68%; B. Yield 610 mg, 75%; C. Yield 665 mg, 82%; D. Yield 455 mg, 56%. ^1^H NMR (400 mHz, DMSO-*d*_6_, *δ*, ppm): 3.50–3.53 (m, 8H, 4 × CH_2_O), 3.87 (t, 4H, 2 × CH_2_O), 4.61 (t, 4H, 2 × CH_2_O), 7.36 (m, 4H, C_20_H_13_), 7.48–7.52 (m, 6H, C_20_H_13_), 7.69–7.78 (m, 6H, C_20_H_13_), 8.29–8-37 (m, 10H, C_20_H_13_), 8.50 (s, 2H, C_2_N_3_H). ^13^C NMR (100 MHz, DMSO-*d*_6_, *δ*, ppm): 49.58, 68.59, 69.60, 120.89, 124.41, 125.47, 126.87, 127.15, 127.50, 127.65, 127.91, 128.01, 128.27, 127.37, 130.16, 130.46, 130.70, 131.53, 134.16, 145.43. Anal. Calcd. For C_53_H_44_N_6_O_3_: C 78.30%, H 5.46%, N 10.34%; Found: C 78.20%, H 5.56%, N 10.36%. ESI-MS, *m*/*z*, (%): Calcd. For [C_53_H_44_N_6_O_3_ + H]^+^ = 813.3548; Found: 813.3541, (100).

## 4. Conclusions

In conclusion, a mechanochemically sustainable and green approach towards PEG-connected bis-4-(PAH)-1*H*-1,2,3-triazoles was developed. This work presents a method for catalyzing an AAC click reaction using bronze microparticles that are formed in situ during the bronze powder ball milling. This heterophase catalyst has been shown to be efficient and inexpensive and suitable for developing green chemistry methods under solvent-free ball-milling conditions. The above-mentioned solvent-free reaction proceeds in a short period of time without the addition of any catalysts or auxiliary ligands required for “click” reactions, with *E*-factors as low as 0.38, and results in the target products with yields up to 96%. This procedure required only the use of a conventional stainless-steel jar, stainless-steel balls, and inexpensive bronze powder as an additive. In addition, this reaction tolerates several types of PAH-based substituents in the 1,2,3-triazole moiety as well as PEG-based coupling partners with various chain lengths.

## Figures and Tables

**Table 1:** Reaction optimization studies and yields of compounds **3a–c**.

No.	Compound	Procedure	Ball Material	Additive	Time, h	Yield, %	*E*-Factor (kg Waste per kg Product)
1	**3a**	A	Cu	Silica gel	3	59	0.86
2	**3a**	B	Cu	Bronze powder	3	91 ^[a]^	0.53
3	**3a**	C	Stainless steel	Bronze powder	3	84	0.65
4	**3b**	A	Cu	Silica gel	3	78	0.38
5	**3b**	B	Cu	Bronze powder	3	65	1.07
6	**3b**	C	Stainless steel	Bronze powder	3	90 ^[a]^	0.49
7	**3c**	A	Cu	Silica gel	3	68	0.62
8	**3c**	B	Cu	Bronze powder	3	75	0.80
9	**3c**	C	Stainless steel	Bronze powder	3	87	0.55
10	**3a**	D	**-**	**-**	16	40	25.54
11	**3b**	D	**-**	**-**	16	70	13.00
12	**3c**	D	**-**	**-**	16	70	12.55

^[a]^ best yield.

**Table 2:** Reaction conditions and yields of compounds **4a**–**b** and **5a**–**b**.

No.	Compound	Procedure	Ball Material	Additive	Time, h	Yield, %	*E*-Factor (kg Waste per kg Product)
**1**	**4a**	A	Cu	Silica gel	3	69	0.90
**2**	**4a**	B	Cu	Bronze powder	3	88 ^[a]^	0.87
**3**	**4a**	C	Stainless steel	Bronze powder	3	80	1.03
**4**	**4b**	A	Cu	Silica gel	3	71	0.54
**5**	**4b**	B	Cu	Bronze powder	3	75	0.80
**6**	**4b**	C	Stainless steel	Bronze powder	3	96 ^[a]^	0.40
**7**	**5a**	A	Cu	Silica gel	3	48	1.27
**8**	**5a**	B	Cu	Bronze powder	3	70	0.92
**9**	**5a**	C	Stainless steel	Bronze powder	3	78	0.72
**10**	**5b**	A	Cu	Silica gel	3	68	0.57
**11**	**5b**	B	Cu	Bronze powder	3	75	0.73
**12**	**5b**	C	Stainless steel	Bronze powder	3	82	0.58
**13**	**4a**	D	**-**	**-**	16	50	19.05
**14**	**4b**	D	**-**	**-**	16	85	10.12
**15**	**5a**	D	**-**	**-**	16	25	37.25
**16**	**5b**	D	**-**	**-**	16	56	14.65

^[a]^ best yields.

## Data Availability

The original contributions presented in this study are included in the article and Supporting Information. Further inquiries can be directed to the corresponding authors.

## Supplementary Materials

The following supporting information can be downloaded at: https://www.mdpi.com/article/10.3390/molecules31020270/s1, Figures S1–S7: ^1^H NMR spectra of the compounds **3**–**5**; Figures S8–S14: ^13^C NMR spectra of the compounds **3**–**5**.

## Abbreviations

The following abbreviations are used in this manuscript:

PEG	Polyethylene glycol
PAH	Polycyclic aromatic hydrocarbon
DMF	Dimethyl formamide
CuAAC	Copper(I)-catalyzed azide–alkyne cycloaddition
NMR	Nuclear magnetic resonance
TMS	Tetramethylsilane
TLC	Thin-layer chromatography
Pyr	Pyrene-1-yl moiety
