# Supplementary material for: Mechano-Induced Synthesis of Polyethylene Glycols α,ω-DiSubstituted with 4-(PAH)-1H-1,2,3-Triazoles in Presence of In Situ-Generated Bronze Microparticles as Catalyst
Source: Molecules. 2026 Jan 13;31(2):270. doi: 10.3390/molecules31020270 (PMC12843628; doi:10.3390/molecules31020270)
Supplement: Supplementary file 1 [file molecules-31-00270-s001.zip › molecules-4029665-supplementary.pdf]

Supporting Information

Mechano-Induced Synthesis of Polyethylene Glycols α,ω-DiSubstituted with 4-(PAH)-1H-1,2,3-Triazoles in Presence of In Situ Generated Bronze Microparticles as Catalyst

Mohammed S. Mohammed, Igor S. Kovalev*, Vadim A. Platonov, Sougata Santra*, Zhuo Wang, Grigory V. Zyryanov and Valery N. Charushin

*Correspondence: ekls85@yandex.ru (I.S. Kovalev); sougatasantra85@gmail.com (S. Santra)

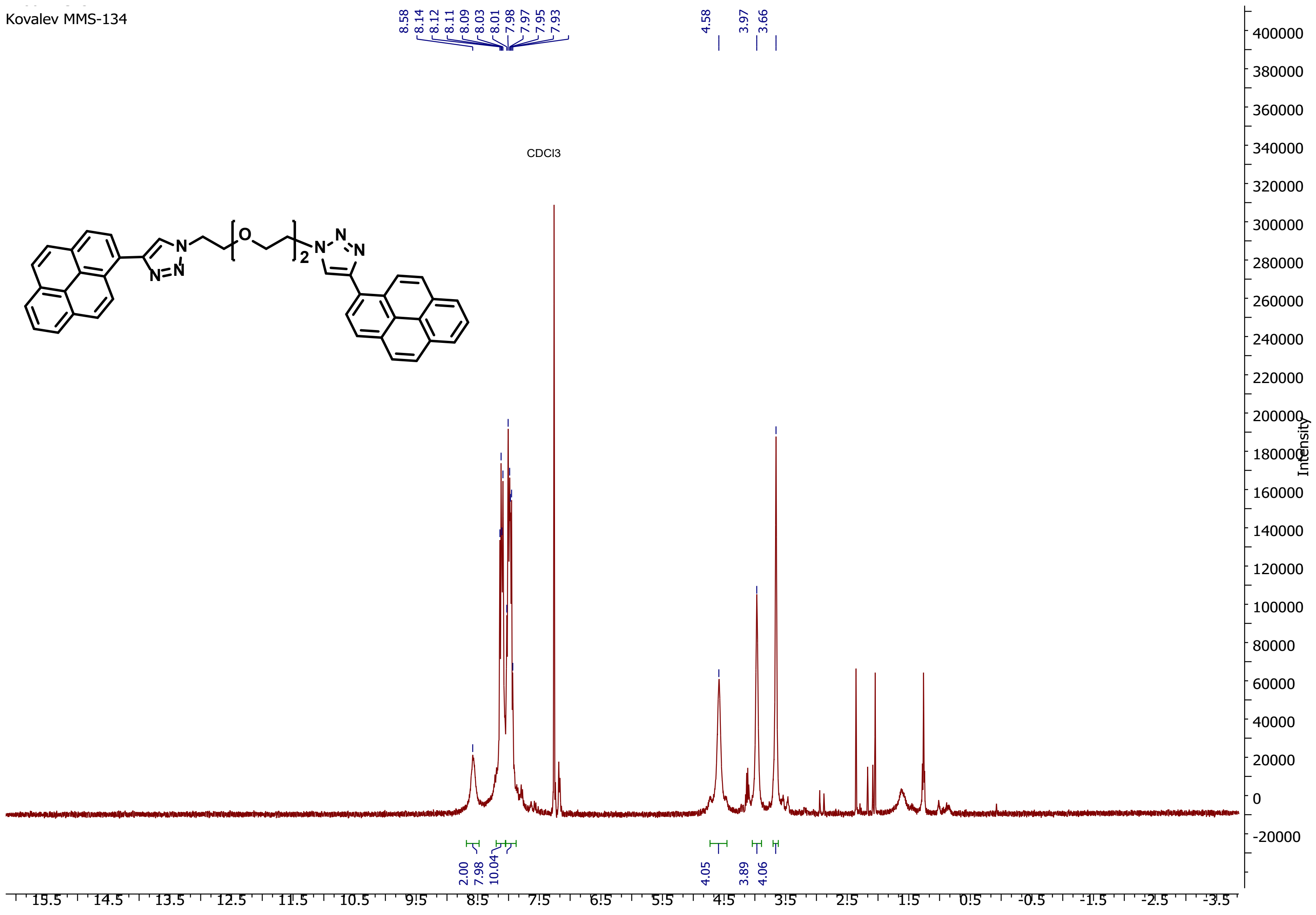

Figure S1. ^{1}H NMR spectrum of compound **3a**

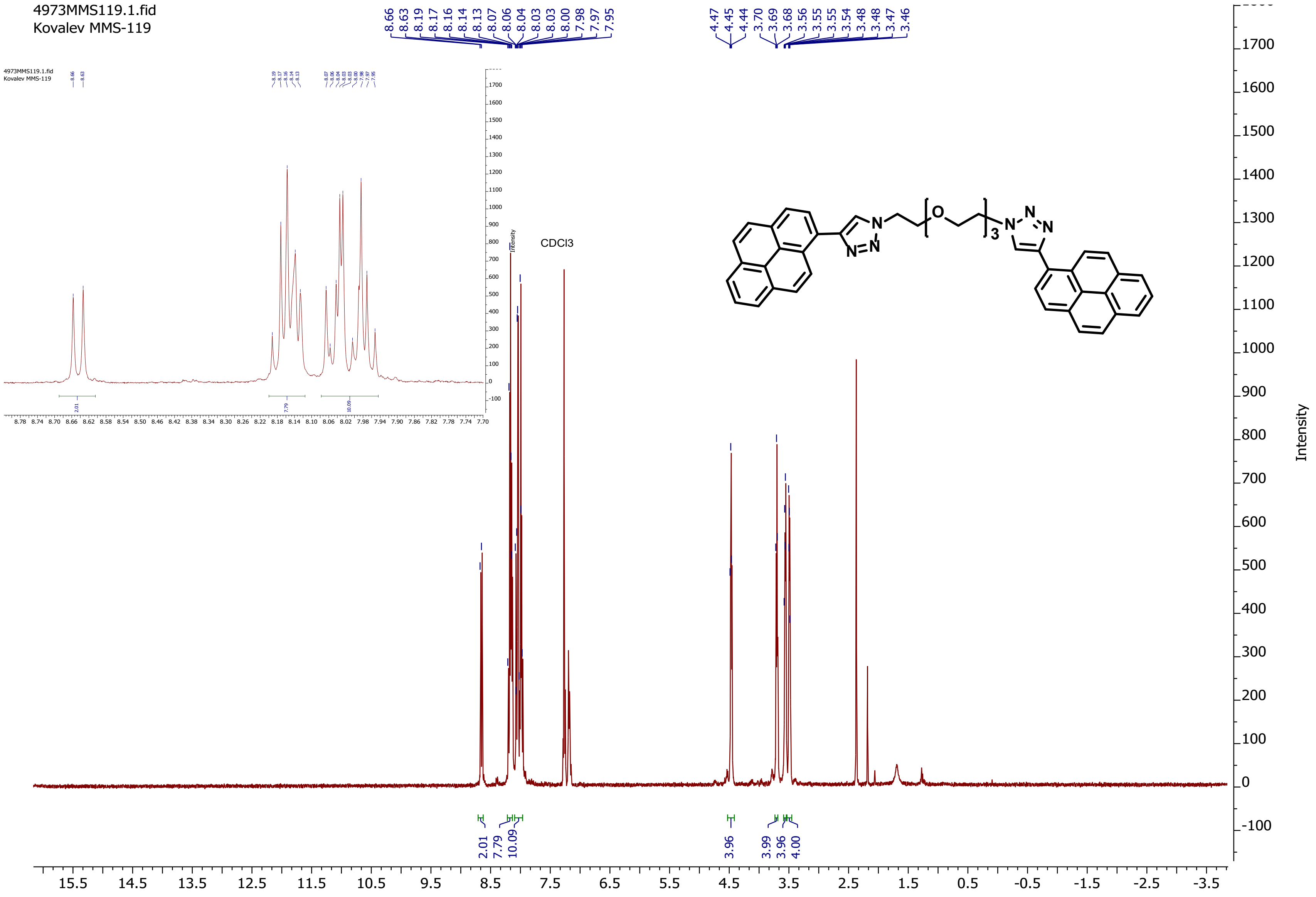

Figure S2. ^{1}H NMR spectrum of compound **3b**

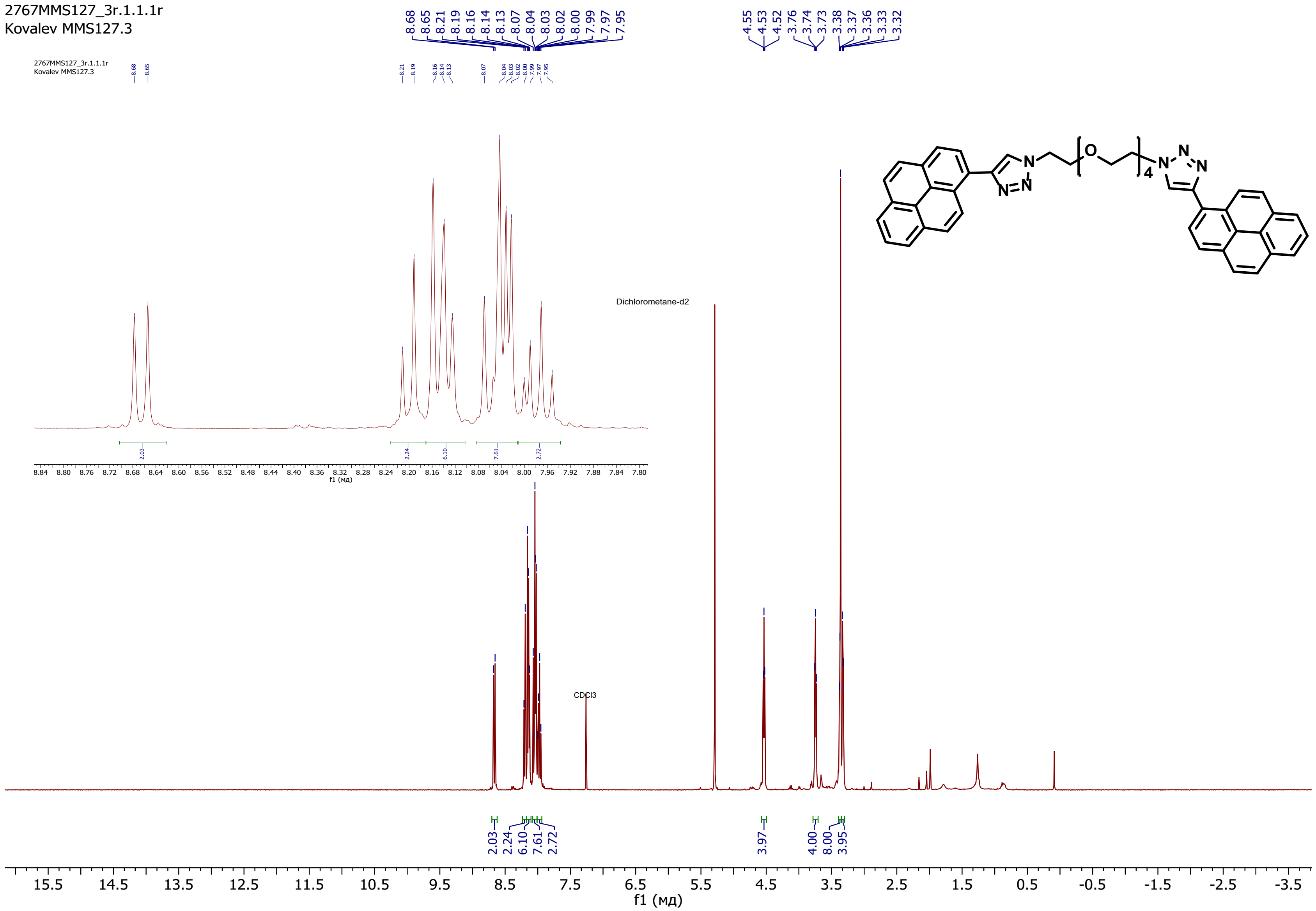

Figure S3. ^{1}H NMR spectrum of compound 3c

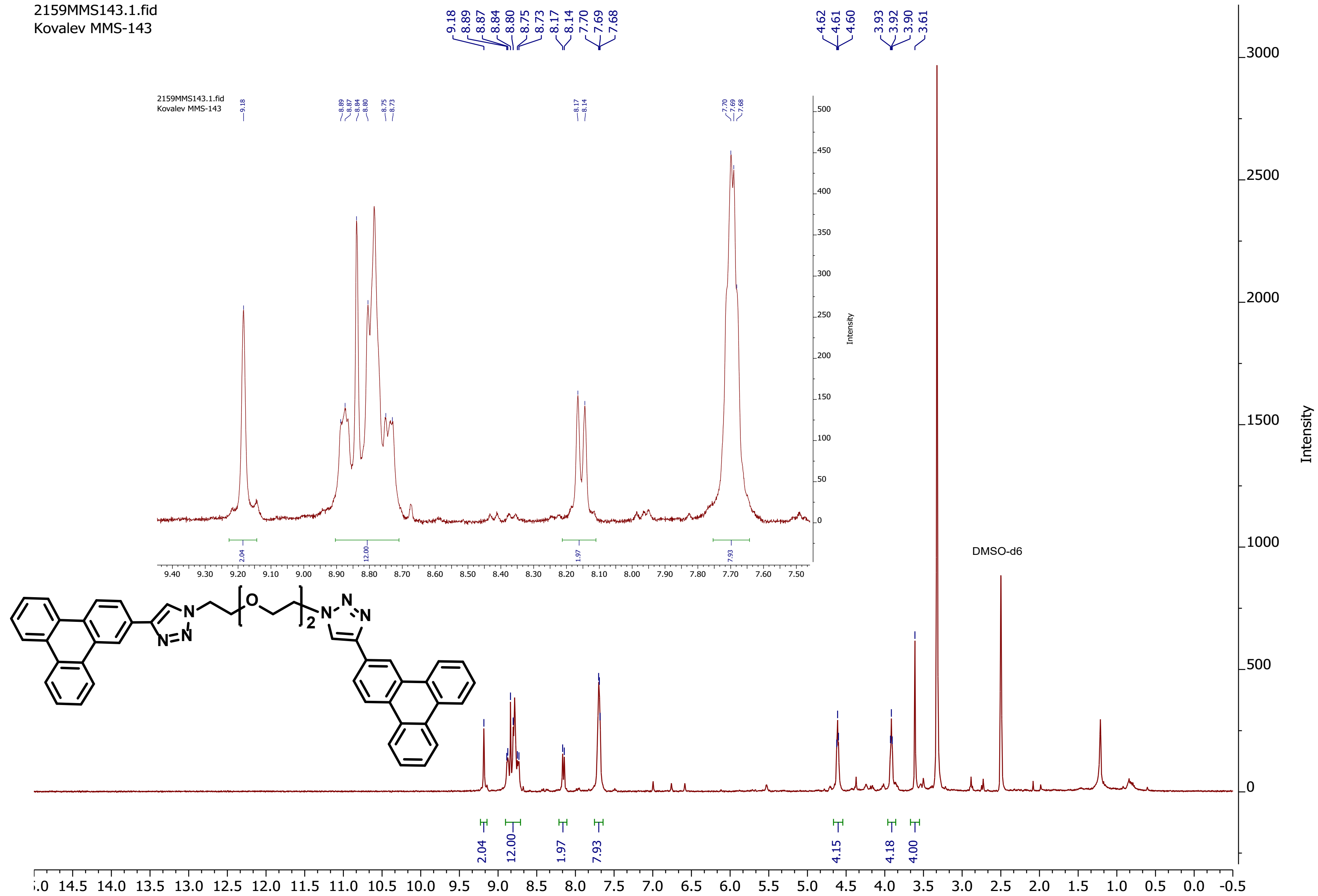

Figure S4. ^{1}**H NMR spectrum of compound 4a**

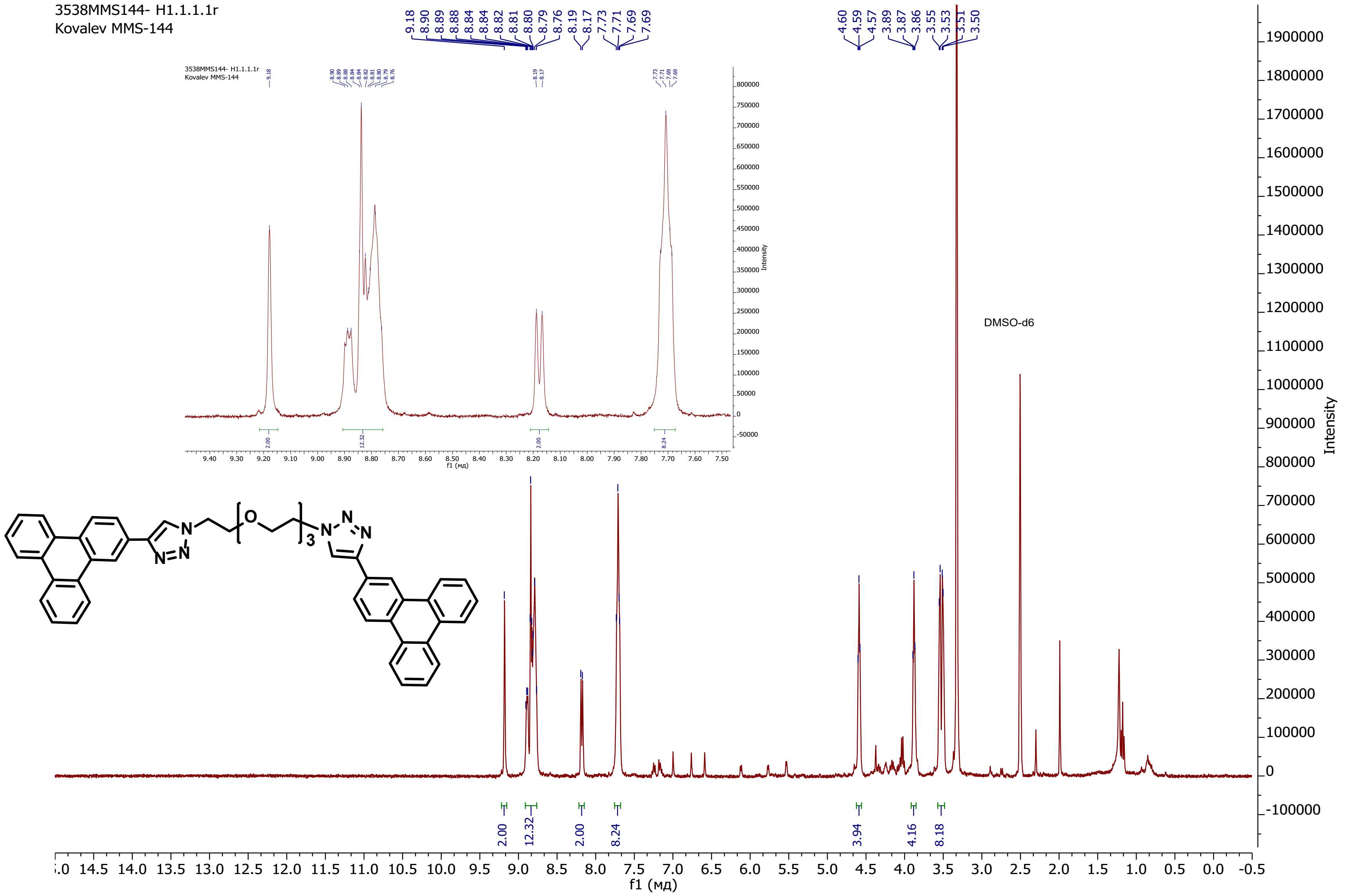

Figure S5. ^{1}H NMR spectrum of compound 4b

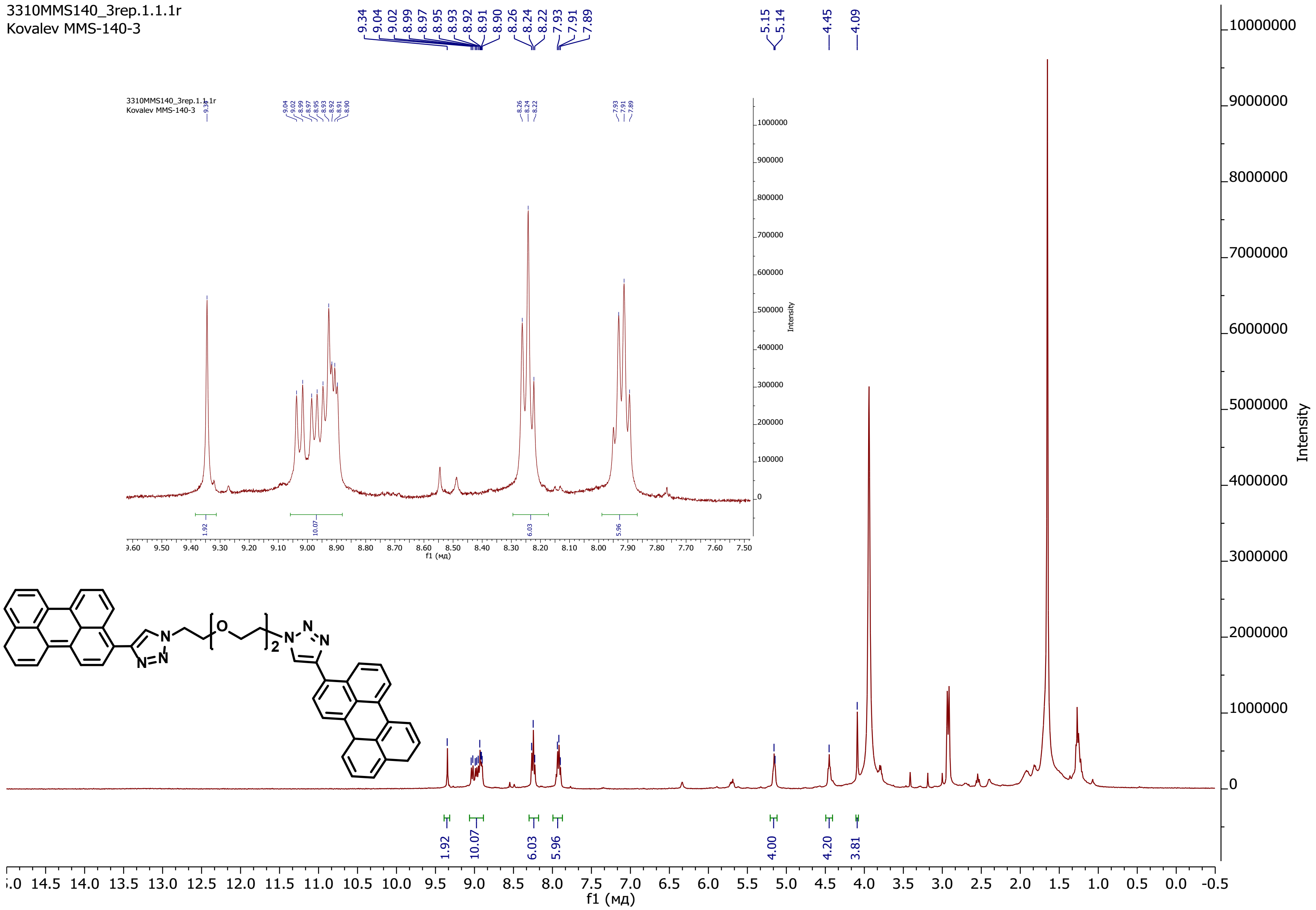

Figure S6. ¹H NMR spectrum of compound 5a in DMF-*d7*

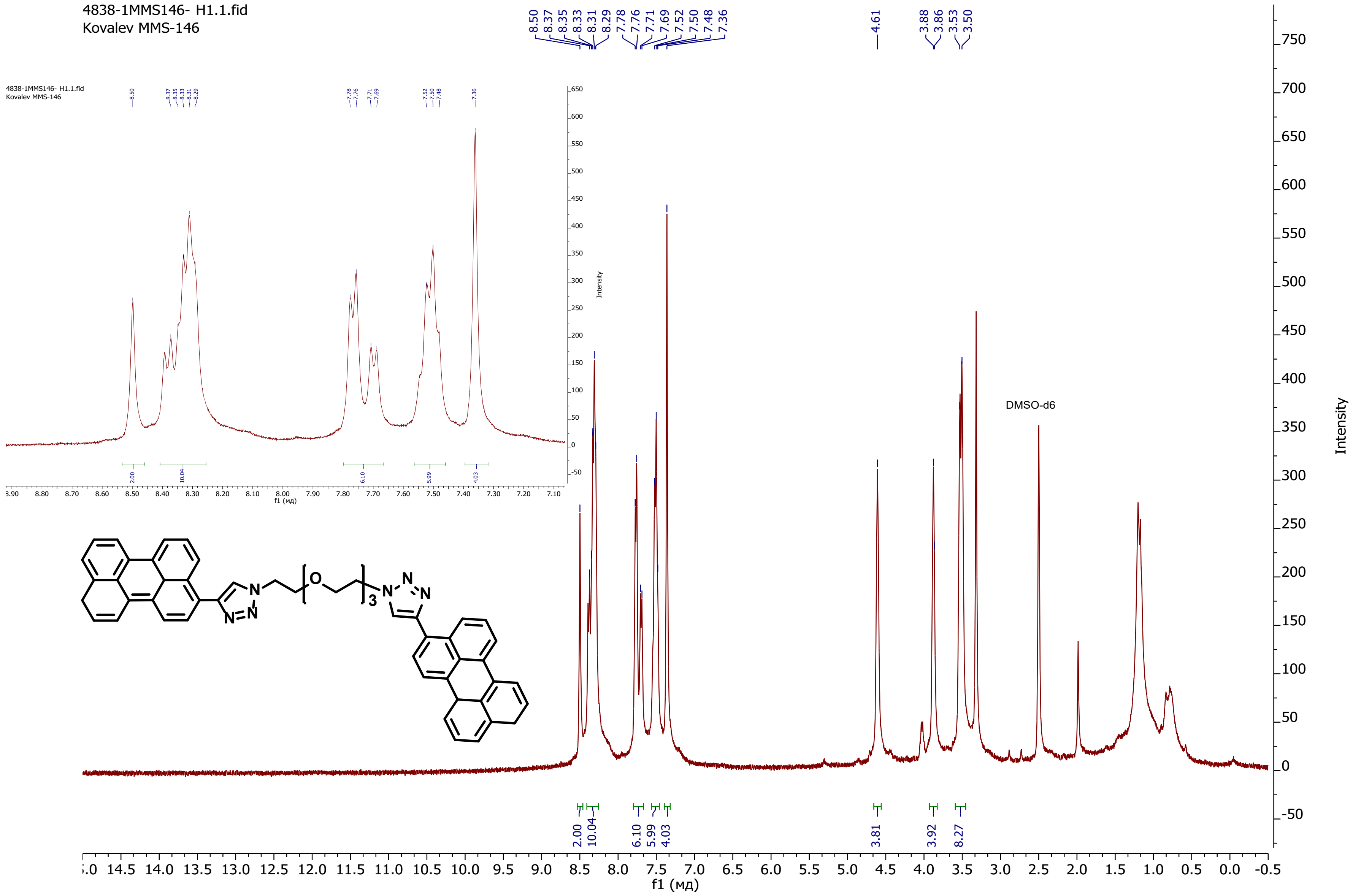

Figure S7. ^{1}H NMR spectrum of compound 5b

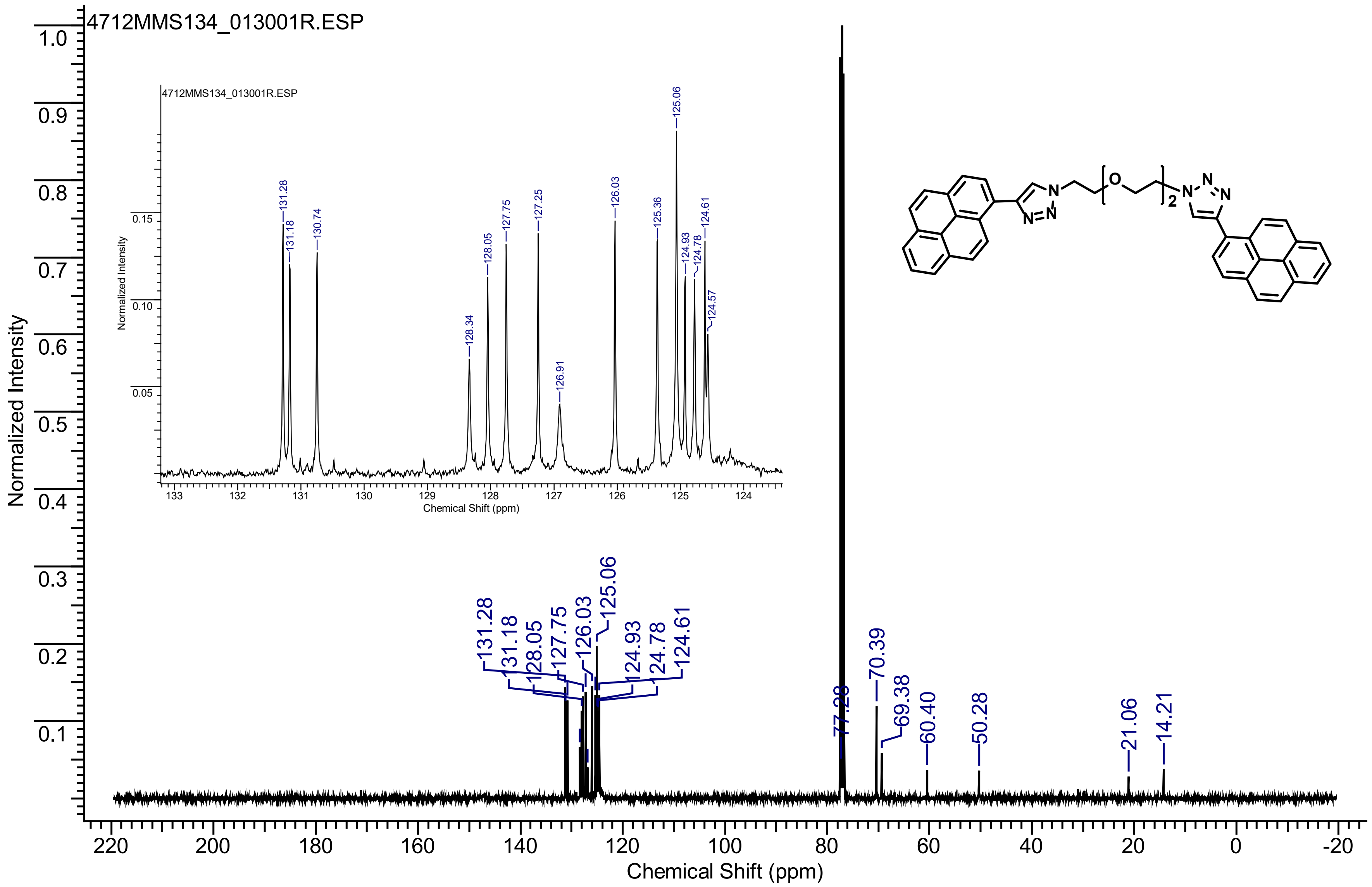

Figure S8. ^{13}C NMR spectrum of compound 3a

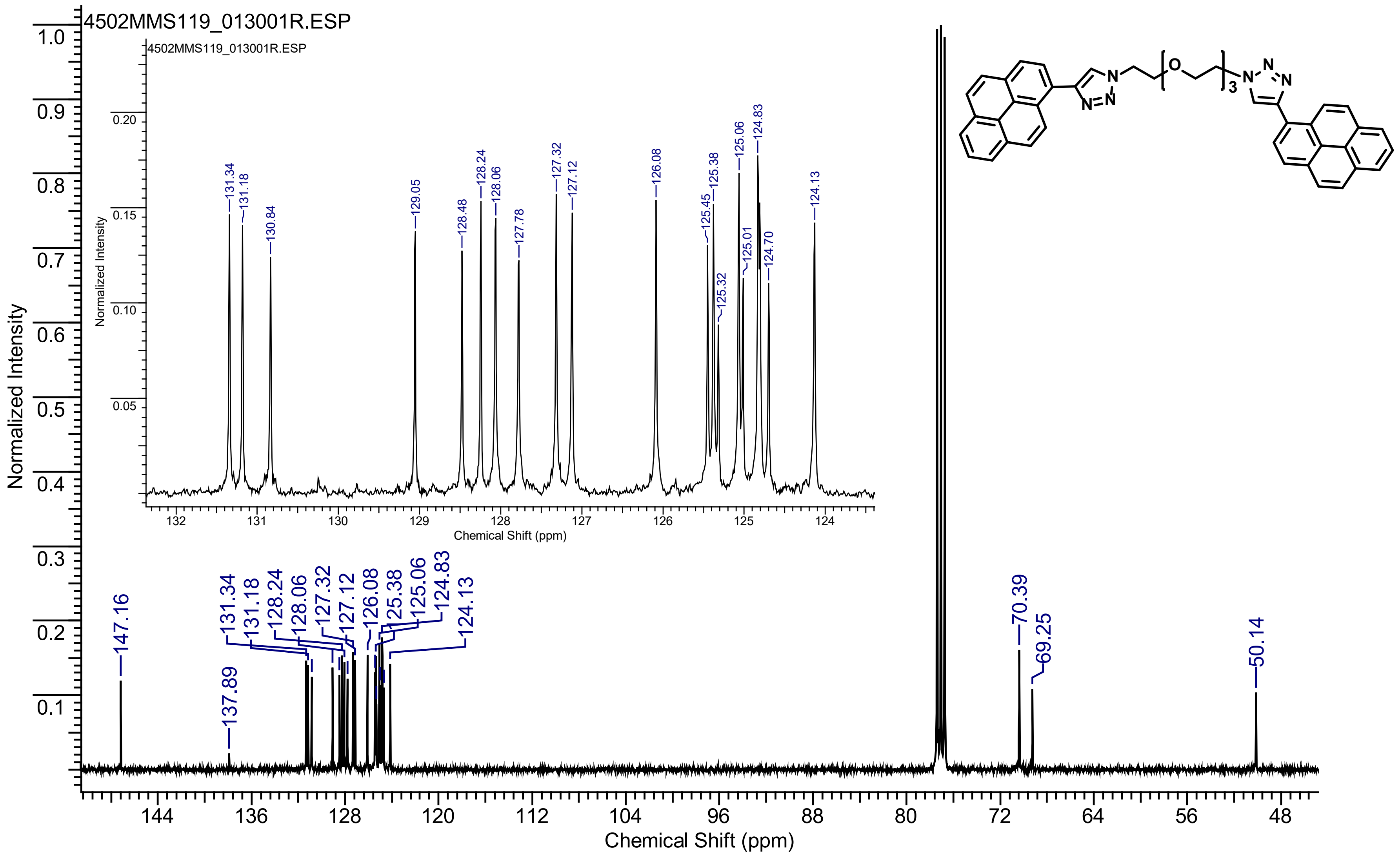

Figure S9. **^{13}C NMR spectrum of compound 3b**

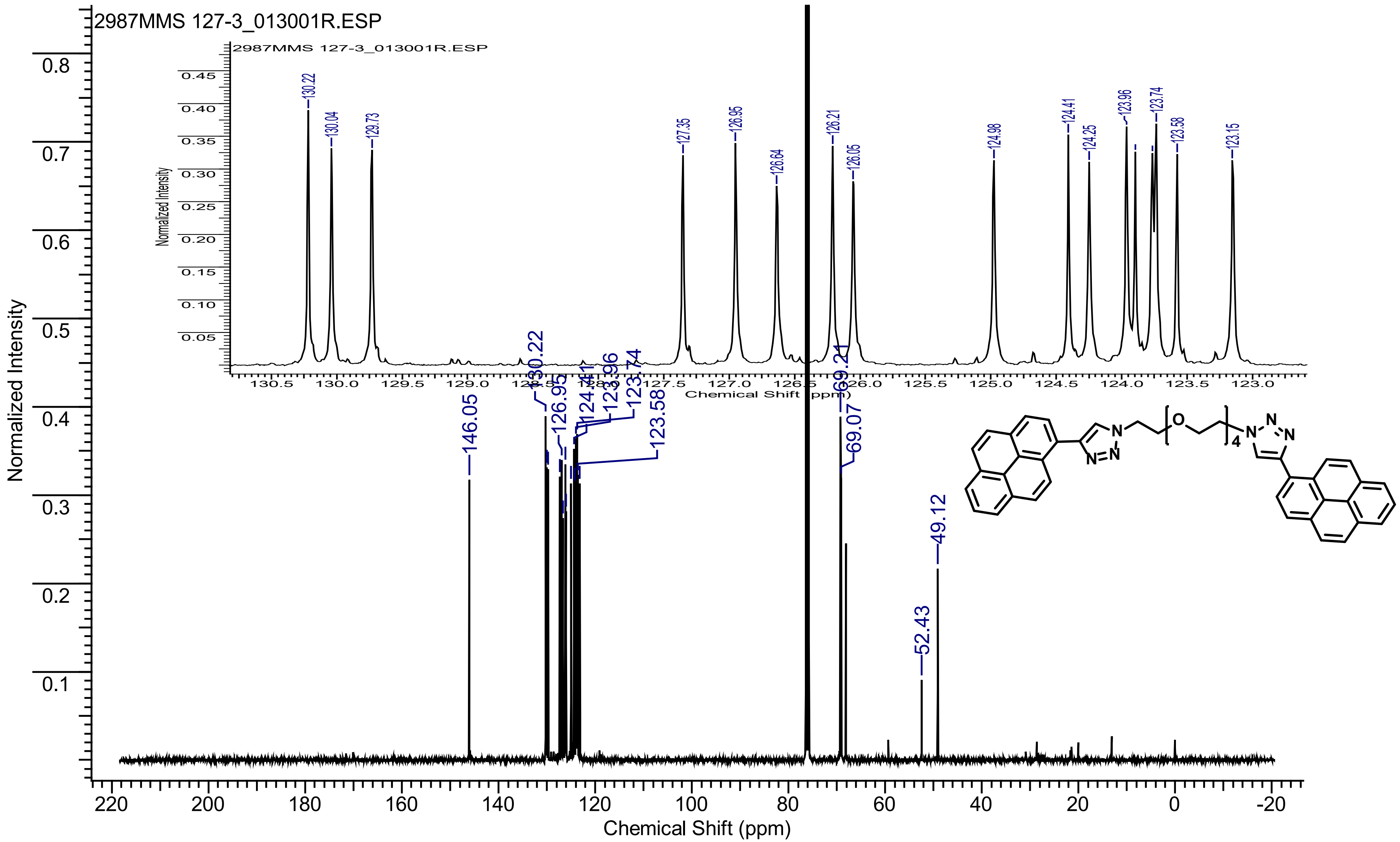

Figure S10. ^{13}C NMR spectrum of compound 3c

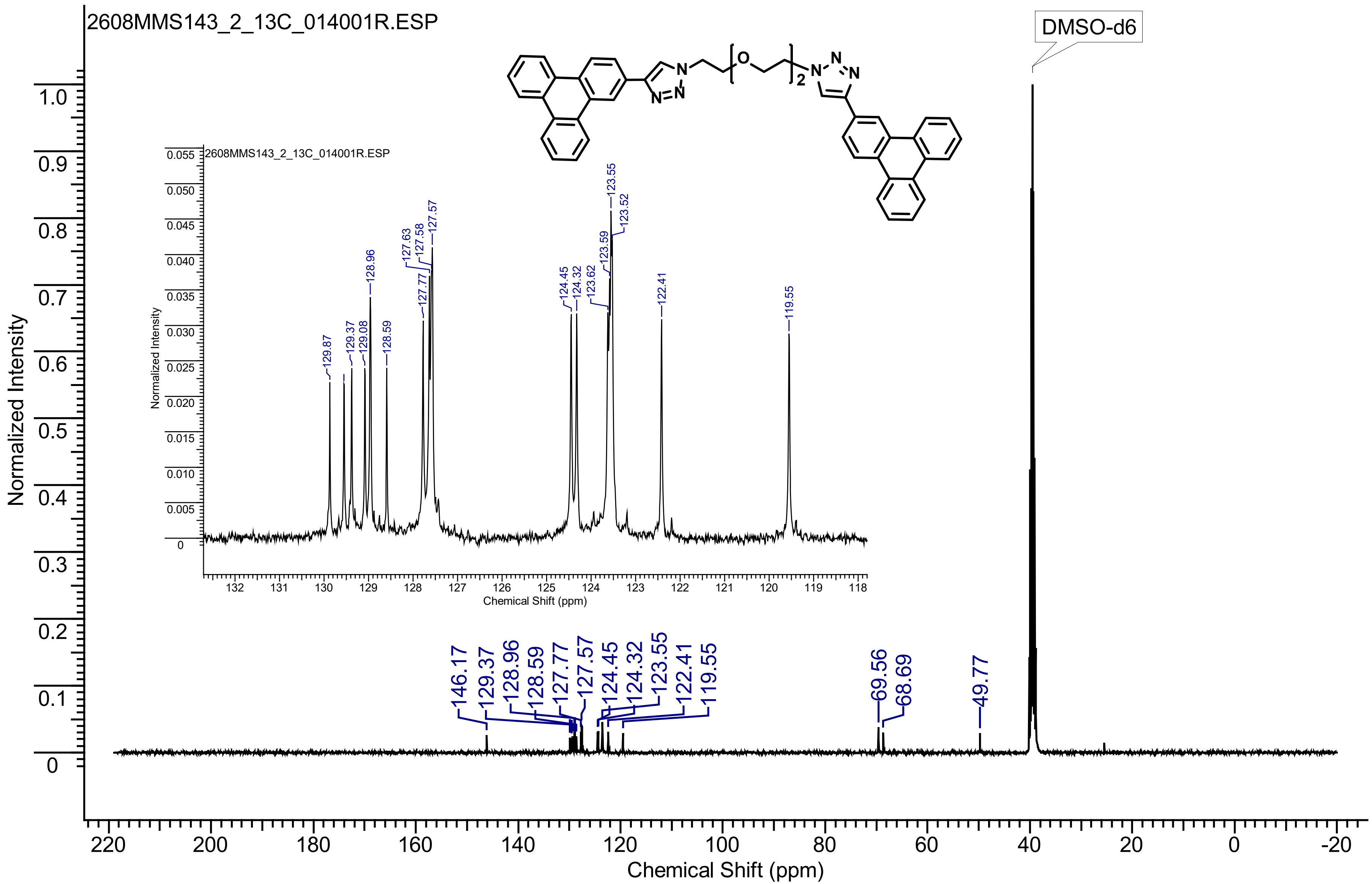

Figure S11. ^{13}**C NMR spectrum of compound 4a**

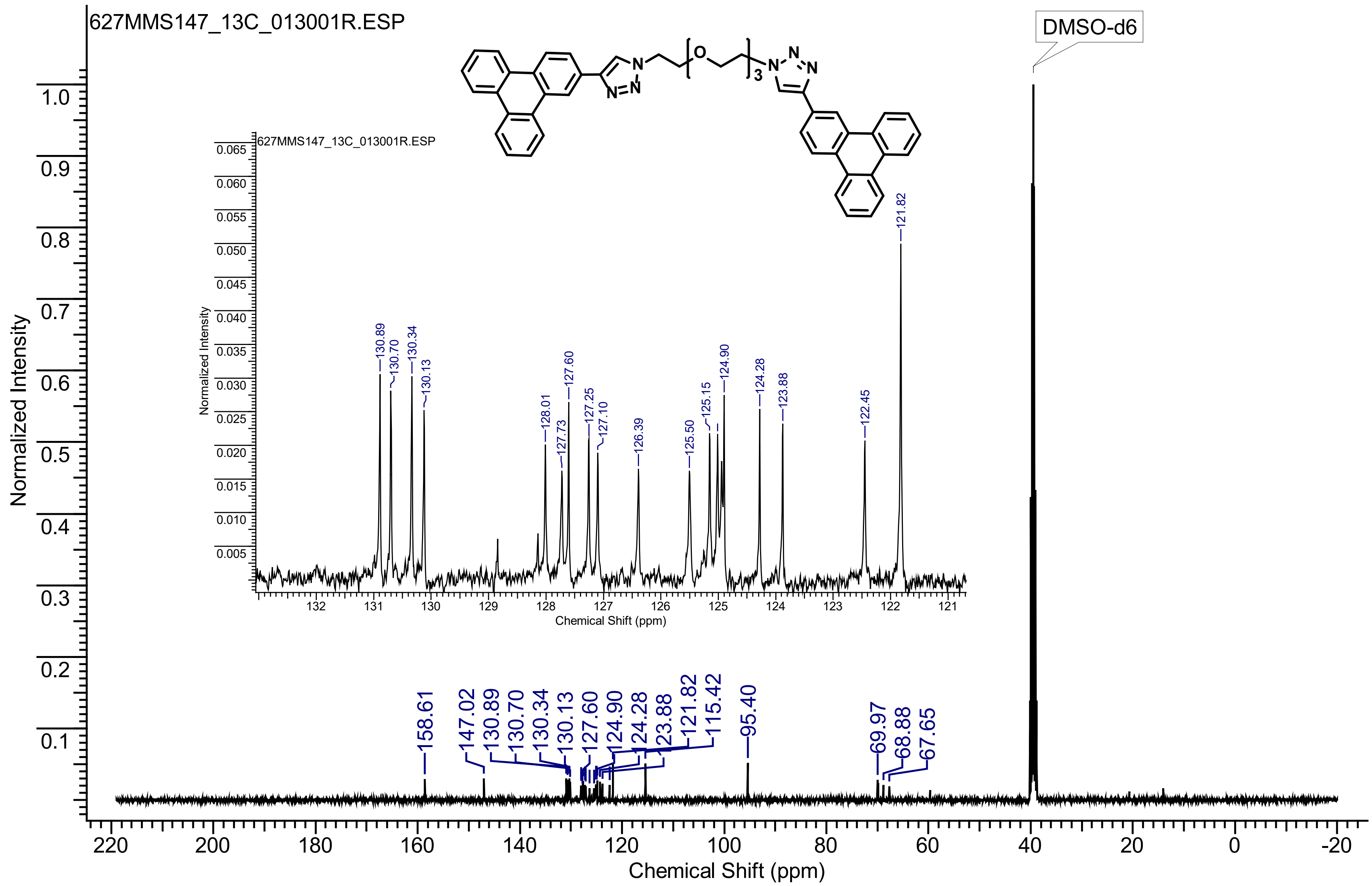

Figure S12. ^{13}C NMR spectrum of compound 4b

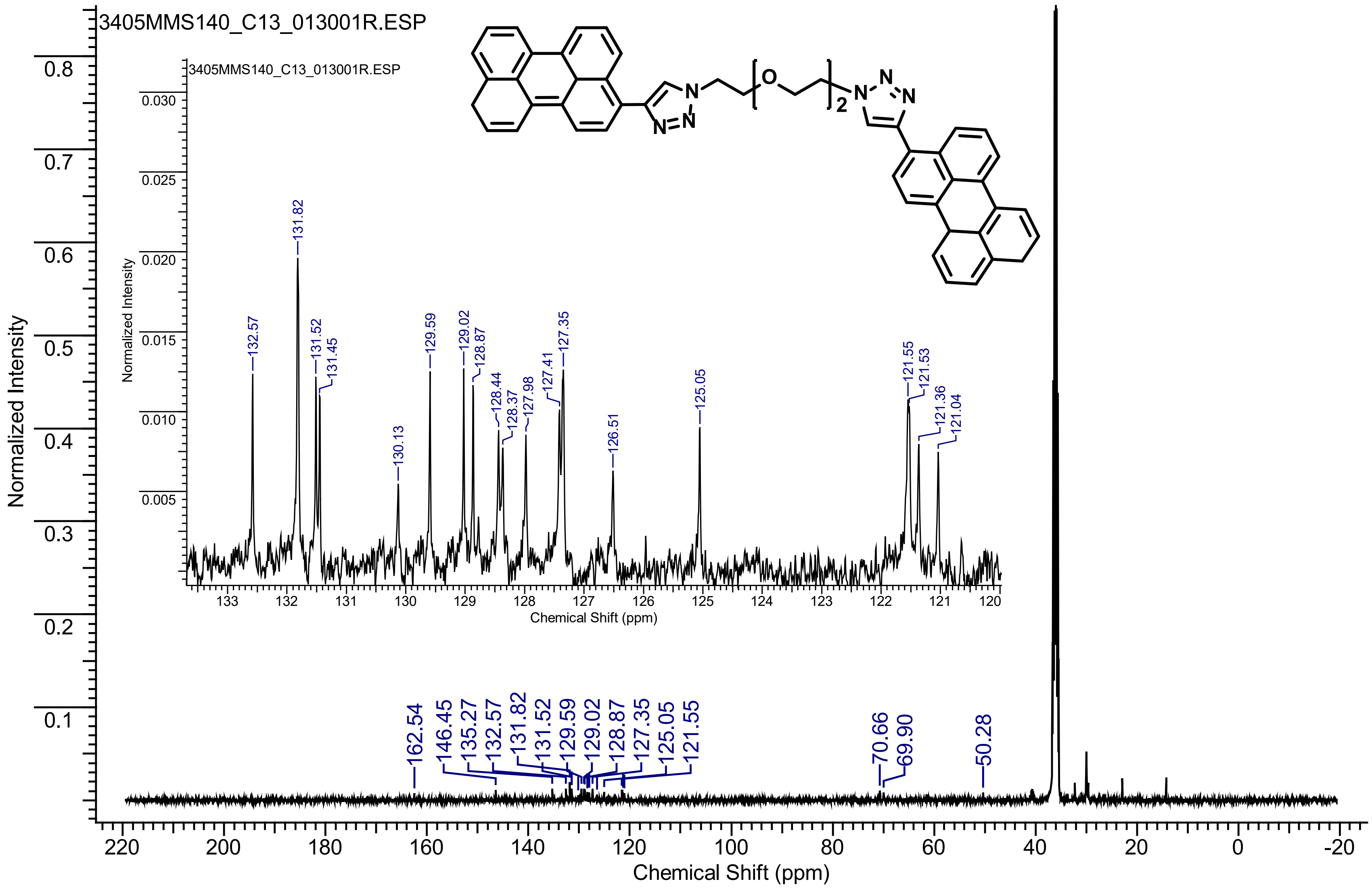

Figure S13. ^{13}C NMR spectrum of compound 5a

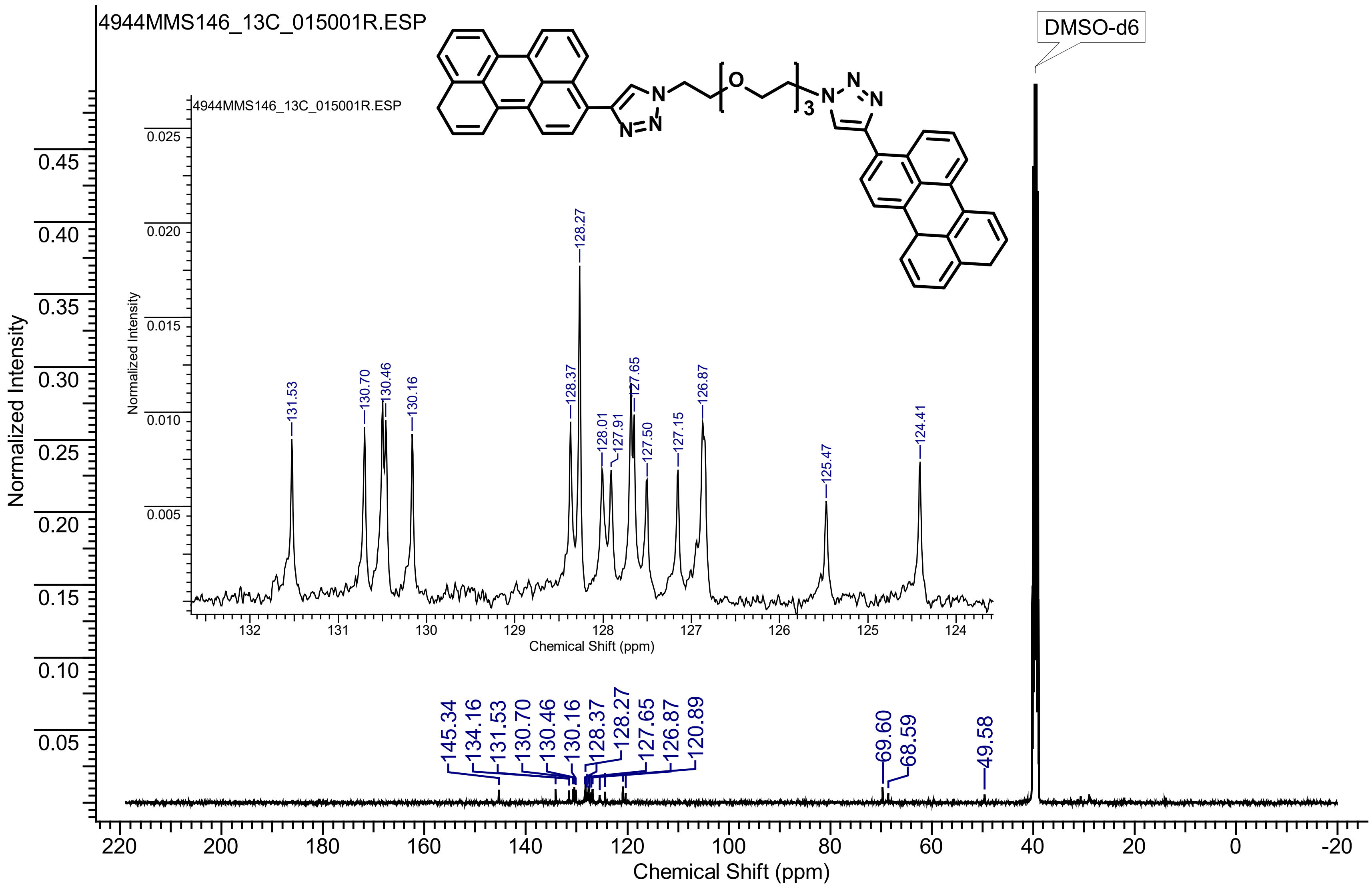

Figure S14. ^{13}**C NMR spectrum of compound 5b**
